# Rapid lake ice structure changes across Swedish lakes puts public ice safety at risk

**DOI:** 10.1007/s13280-024-02067-8

**Published:** 2024-08-20

**Authors:** Kevin Vikström, Gesa Weyhenmeyer, Ellinor Jakobsson, Mark Peternell

**Affiliations:** 1 Department of Ecology and Genetics/Limnology, Uppsala University, Evolutionary Biology Centre, EBC Norbyvägen 14-18, 752 36 Uppsala, Sweden; 2 Department of Earth Sciences, University of Gothenburg, Medicinaregatan 19, 41390 Gothenburg, Sweden

**Keywords:** Global warming, Ice structure, Ice thickness, Lake ice, Public safety

## Abstract

Lakes are rapidly losing ice under global warming, but little is known about ice structure changes. Ice structure is a key regulator of ice stability and thus safety, affecting activities on ice. Here, we analysed spatial and temporal variations in ice structure across 21 Swedish lakes, spanning from 55 to 69 °N, and over five decades. We found regional differences in ice structure, with fastest changes occurring in southern Sweden. The stable clear ice layer was particularly sensitive to warming, showing a rapid decline. The number of days when temperatures exceeded the freezing point during the ice cover period was identified as a strong driver for how ice was structured. Since there is a high risk for increased occurrences of unsafe ice conditions under predicted air temperature changes, we recommend re-establishing ice structure monitoring programmes, informing society on the increased risks of being on ice and including ice structure to safety guidelines.

**Supplementary Information:**

The online version contains supplementary material available at 10.1007/s13280-024-02067-8.

## Introduction

Seasonal ice cover is a common and important phenomenon for lakes of the Northern Hemisphere (Benson et al. 2012; Newton & Mullan 2021). Lake ice provides a large variety of ecosystem services, ranging from winter transportation (Prowse et al. 2011), recreation (Knoll et al. 2019), fishing (Pierce & Cook 2000; Van Assche et al. 2013) to the regulation of the hydrological cycle (Kirillin et al. 2012; Cavaliere et al. 2021). All these ecosystem services from lake ice are presently endangered as long-term ice observations clearly demonstrate a rapid decline in the extent and duration of ice cover on lakes around the Northern Hemisphere under global warming (Magnuson et al. 2000; Newton & Mullan 2021; Sharma et al. 2021). Rapid lake and river ice cover loss across the Northern Hemisphere has been confirmed by analysing satellite images (e.g. Pour et al. 2017; Mäkynen et al. 2020; Higgins et al. 2021), applying microwave remote sensing (e.g. Murfitt & Duguay 2021) and through modelling efforts (e.g. Duguay et al. 2003; Elo 2006; Robinson et al. 2021). As air temperatures continue to increase, many periodically ice-covered lakes will become permanently ice-free (Sharma et al. 2019), with fastest changes occurring in the warmest geographical regions (Weyhenmeyer et al. 2011).

**Fig. 1:**
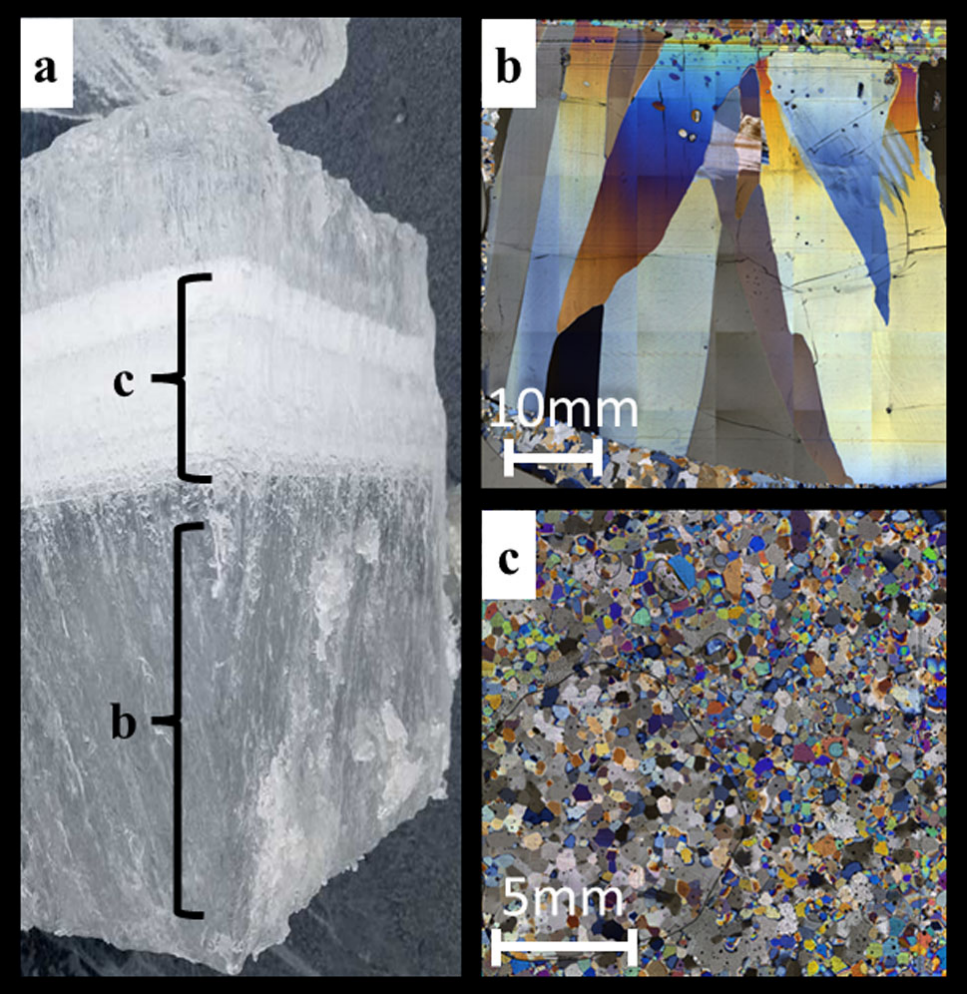
**a** Example of an ice core with clear and white ice and microphotographs of its internal ice microstructures. **b** The up to 5 cm long, tabular crystals in the clear ice grew with their long axes parallel to the lake surface, whereas white ice **c** consists of < 5 mm orbicular grains with an approximately random orientation

The consistency in observations of lake ice cover loss under the ongoing global warming trend is striking, but how lake ice will change in structure in a warmer world remains unclear. Ice structure refers to a variety of variables of which the total ice thickness, and the thickness of clear and white ice is among the most important variables to determine ice stability and safety (Leppäranta 2015; Weyhenmeyer et al. 2022). The first ice to form on lakes during the winter period is clear ice which develops from the initial nucleation of supercooled water (Michel and Ramseier 1971). Clear ice commonly exhibits lower amounts of impurities such as air bubbles and consists of large crystals with a clear direction of growth (Fig. 1a) giving clear ice a strong bearing capacity. Further growth of ice is driven by cooling of surface lake water as heat escapes through the ice sheet to balance out the colder air temperatures (Leppäranta 2009). If heat transfer and the consequent ice formation are disturbed by, for example, air temperatures well above the freezing point or by snow on ice, the ice structure can change and white ice forms. White ice typically consists of smaller, granular crystals showing randomised directions of growth with a higher amount of impurities, homogenously distributed or concentrated in layer or lenses (Fig. 1b). The bearing strength of white ice is lower than that of clear ice and varies alongside ice temperature. White ice bearing strength is reported to be 1.5–5 kg cm^−2^, while clear ice varies from 10 to 20 kg cm^−2^ depending on temperatures (Barrette 2011). The formation of white ice is promoted when air temperatures first rise above the freezing point and then fall below the freezing point or by flooding events occurring due to the weight of snow on ice. Such conditions turns the ice surface and snow into slush which then refreezes, forming white ice (Leppäranta 2015, 2023).

A variety of models exist to determine the allowable load on ice (e.g. Gold 1971; Dinvay et al. 2019; Fitzgerald & Janse van Rensburg 2023) of which Gold’s equation is a widely used model due to its simplicity. Recently, Gold’s equation has further been developed to better account for white ice conditions (Weyhenmeyer et al. 2022). When ice bearing capacity becomes low, the risk of fatal winter drownings by people falling through ice increases. For example, results from a recent survey indicated that most fatal winter drownings occur when mean winter air temperatures approach zero °C (Sharma et al. 2020). In lake-rich countries like Sweden (100 000 lakes > 0.01 km^2^, SMHI 2009), an average of eleven people die every year by falling through ice (average during 2020–2023, Swedish Life Saving Society 2023). However, reports of non-fatal accidents are lacking, which for Sweden are probably manifold considering the popularity of wintertime activities.

At present, ice safety guidelines vary but 15 cm of ice is generally considered safe for walking and skating on ice while 25 cm thickness is generally seen as safe for snowmobiling (Minnesota Department of Natural Resources 2022; Canadian Red Cross 2014; Issäkerhetrådet 2012; Swedish Snowmobile Owners State Organisation 2012). Although total ice thickness gives some indications on the allowable load on ice, all guidelines strongly recommend testing the stability of ice before performing any activity on ice. Literature reports total ice thickness declines over the past decades (Leppäranta & Kosloff 2000; Karetnikov & Naumenko 2008; Hawley et al. 2018). These studies, however, fail to consider changes in ice structure. Since the thickness of the clear ice layer plays a key role for ice safety, we provide here for the first-time trends over time in total ice thickness and clear and white ice thickness across 21 Swedish lakes spanning over 13 degrees of latitude. The following three questions were addressed in detail: (1) How have ice-conditions across Swedish lakes changed over time? (2) Where in Sweden was observed ice structure changes most prominent? (3) What consequences do lake ice structure changes have for public lake ice safety? We hypothesized that (a) lake ice safety in Sweden has significantly decreased during the past decades, mainly due to a significant decrease in the total and clear ice thickness, and (b) the number of days when air temperatures remain above the freezing point during the ice cover period is significantly related to the thickness of clear and white ice. To test the two hypotheses, we used data on total ice thickness as well as clear and white ice thickness from 21 lakes across Sweden. We then discuss the results from a public safety perspective for guidance to society and stakeholders.

## Materials and methods

### Data

#### Lake ice data

**Fig. 2:**
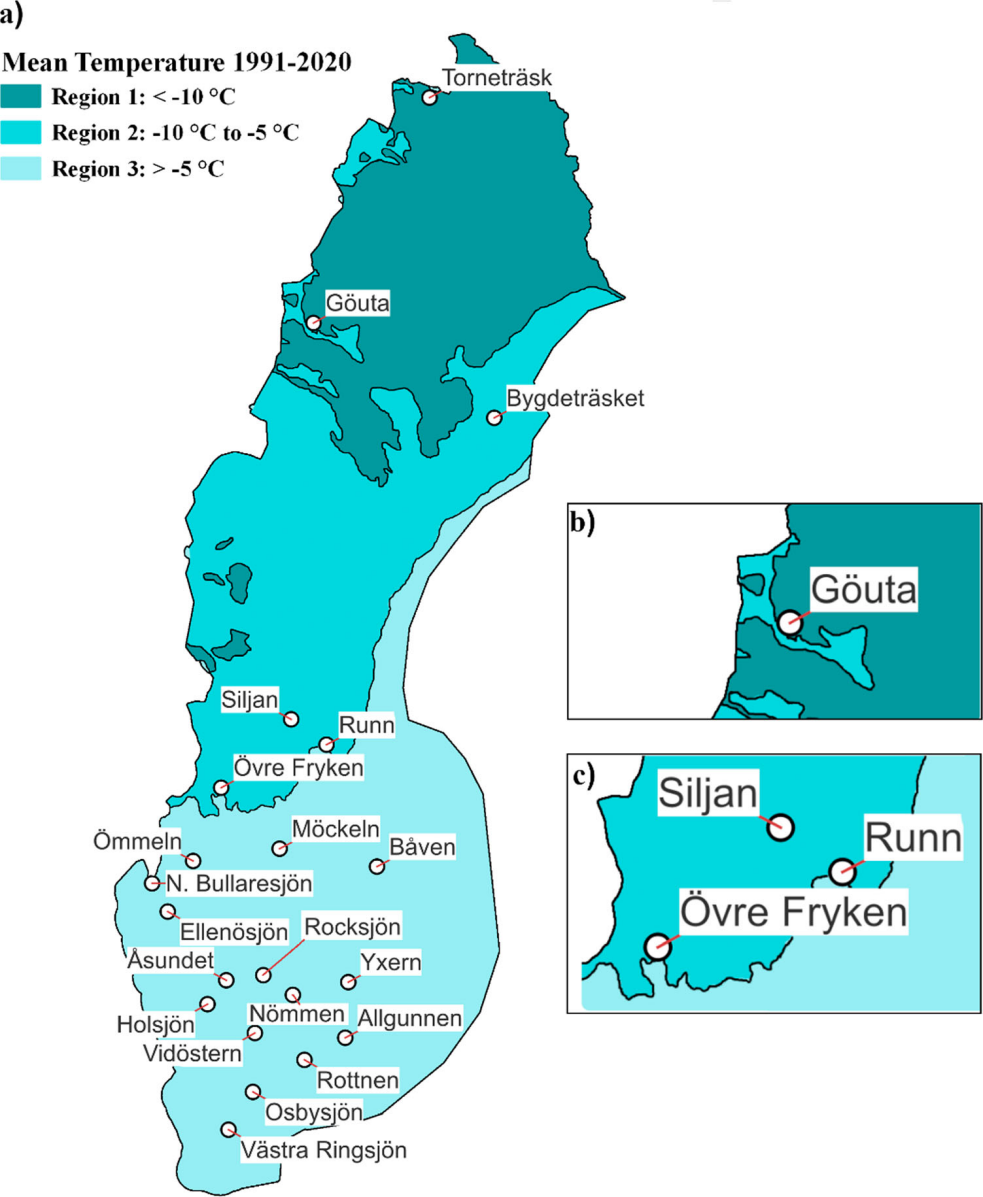
Map showing the name and the location of the 21 lakes and the three regions used in this study as a gradient where, region 1 is light blue and represents mean temperatures < − 10 °C, region 2 mid tone blue and represents temperatures between − 5 and − 10 °C and region 3 dark blue representing temperature > − 5 °C. The geographical region division is based on long-term (1991–2020) mean air temperatures in January (see methods)

Lake ice monitoring data were available from 1950 to 2012 for a total of 45 lakes from the Swedish Meteorological and Hydrological Institute (SMHI). In 2012, this detailed ice monitoring programme was terminated, and many lakes lacked data in the beginning or the end of the time series. Therefore, we restricted our data analyses to the time period of 1960 to 2009 and a minimum of 250 ice observations during those five decades. Based on those criteria, 21 out of the original 45 lakes remained. Osbysjön, Ömmeln and Ellensjön lacked ice thickness data in the early 1960’s but since they fulfilled the criteria of having more than 250 ice observations they were included in this study (Fig. 2, Table 1). Whenever we refer to a year in this study, we included the period November–December from the previous year to capture the entire ice cover period.

**Table 1:** Lake coordinates (decimal degrees), the total number of observations within the dataset after applying the temporal and observational criterion, the years of data, the assigned regions (Region 1: < − 10 °C, Region 2: − 5 to − 10 °C, Region 3 > − 5 °C) and temperature from the normalised mean January winter temperatures (°C) is reported with temperatures in brackets after the region name. The data is organised from the warmest to coldest region. Additionally, lake surface area and mean depths are reported for each lake

Lake	Latitude	Longitude	Years of data	N	Region	Lake surface area (km^2^)	Mean lake depth (m)
Rocksjön	57.771	14.182	1960–2009	297	Region 3 (− 1 °C)	0.324	11.3
Ellenösjön	58.505	11.953	1962–2009	362	Region 3 (− 1 °C)	2.9	7*
Osbysjön	56.353	13.986	1961–2009	359	Region 3 (− 1 °C)	4.68	1.9
Holsjön	57.405	12.938	1960–2009	341	Region 3 (− 2 °C)	5.41	4.2
Ömmeln	59.131	12.497	1961–2009	346	Region 3 (− 3 °C)	6.85	26
N. Bullaresjön	58.838	11.559	1960–2009	445	Region 3 (− 1 °C)	6.88	20.6
Allgunnen	57.010	16.017	1960–2009	344	Region 3 (− 1 °C)	13.2	3.5
Västra Ringsjön	55.893	13.472	1960–2009	378	Region 3 (0 °C)	14.4	2.7
Yxern	57.684	16.105	1960–2009	540	Region 3 (− 2 °C)	14.9	8.1
Nömmen	57.534	14.852	1960–2009	665	Region 3 (− 2 °C)	15.4	4.7
Rottnen	56.746	15.111	1960–2009	349	Region 3 (− 2 °C)	32.5	4.5
Åsundet	57.701	13.346	1960–2009	308	Region 3 (− 2 °C)	32.7	12.7
Vidöstern	57.068	14.010	1960–2009	315	Region 3 (− 2 °C)	42.6	4.6
Möckeln	59.303	14.531	1960–2009	619	Region 3 (− 3 °C)	46.1	2.8
Båven	59.074	16.823	1960–2009	617	Region 3 (− 3 °C)	64.2	9.4
Bygdeträsket	64.415	20.455	1960–2009	1078	Region 2 (− 8 °C)	29.3	13.7
Övre Fryken	60.001	13.107	1960–2009	617	Region 2 (− 5 °C)	41.9	93
Runn	60.533	15.665	1960–2009	1006	Region 2 (− 5 °C)	63.5	8.3
Siljan	60.869	14.796	1960–2009	852	Region 2 (− 5 °C)	293	27.8
Göutan	65.665	15.412	1960–2009	1241	Region 1 (− 10 °C)	31.6	17.2
Torneträsk	68.338	19.259	1960–2009	1047	Region 1 (− 11 °C)	332	51

*Maximum depth reported as mean depth value was lacking

From the lake ice monitoring dataset, total ice thickness and the thickness of the clear ice layer were used. The white ice layer thickness was determined by subtracting clear ice thickness from the total thickness of ice. Some additional variables were occasionally reported, i.e. thickness of snow, snow ice, slush on ice, thickness of upper ice layer, thickness of lower ice as well as water on top of ice, but since those additional variables were incomplete, they were not further considered. We used reported ice-on and ice-off dates, when available, to determine when air temperatures remain above the freezing point during the reported ice cover period. Ice-on in the dataset is defined as the first time a lake is covered by ice for three consecutive days, while ice-off is defined as the first time when the lake surface area is ice-free with exceptions of small free floating ice sheets (SMHI 1999).

#### Air temperature data

To test our second hypothesis we used gridded air temperature data from the same grid in which a lake was located in. The gridded air temperature data had a resolution of 2.5 km and were available as daily mean air temperatures since 1961 from the SMHIGridClim model (accessed 11/09/2022 SMHI 2022a). For each lake, when ice-on and ice-off dates were available, we calculated the number of days when daily mean air temperatures were above the freezing point during the ice cover period for each lake and year. Ice-on and ice-off dates were not always reported, reducing the number of days with T > 0 °C that could be calculated to 828 observations from 1020 yearly ice structure observations.

### Defining bearing capacity as an ice safety estimate

Ice safety is a function of the bearing capacity of ice. Ice safety guidelines agree that a total ice thickness of 15 cm is safe to walk on ice depending on (Minnesota Department of Natural Resources 2022; Issäkerhetrådet 2012; Canadian Red Cross 2014). These recommendations are commonly based on Gold’s (1971) original equation:

1 $$P=A*{H}^{2}$$

where *P* is the allowable load (in kg), *H* is the ice thickness (in cm) and *A* is the bearing strength for ice, ranging from 3.5 to 17.5 kg cm^−2^ depending on the structure of ice. To avoid overestimating allowable loads most guidelines refer to the lower estimate of 3.5 kg cm^−2^ for bearing strength (Minnesota Department of Natural Resources 2020).

Gold’s equation has limitations (Fitzgerald & Janse van Rensburg 2023) but its simplicity and easily measured input variables makes the equation powerful and has therefore been further modified to account for the lower bearing capacity of white ice (Weyhenmeyer et al. 2022):

2 $$P=\frac{A*{H}^{2}}{2}\left(1+\frac{100-\%white\,ice}{100}\right)$$

where *P* is the allowable load (in kg), *H* is the ice thickness (in cm), *A* is the bearing strength for ice, corresponding to 3.5 kg cm^−2^ (see above) and %white ice is the proportion of white ice in the ice layer in percentage. In this study, we used the modified Gold’s equation to estimate the amount of weight an ice cover can hold before it breaks.

### Data analyses

As a first step, an overview of spatial and temporal variations of total ice thickness, clear and white ice thickness, and estimates of allowable loads on ice was performed. For the overview, lake data was grouped into regions and decades. The division into regions was based on mean air temperature data for the month of January over the latest 30-year reference period 1991–2020, with 5 °C intervals (Fig. 2, Table 1). January was chosen as the middle month of the meteorological winter in Sweden defined as November-March (SMHI 2023). The air temperature data were taken from the Swedish Meteorological and Hydrological Institute (SMHI 2022b). Using 5 °C increments resulted in three regions in a north to south gradient. The first region was the northern region with temperatures below − 10 °C and covered two lakes. The second region, referred to as the central region had temperatures between − 5 and − 10 °C and included four lakes. The remaining fifteen lakes were grouped into a third region which is the southernmost area of Sweden and had temperatures larger − 5 °C. The mean temperature of the lakes within the central region differed from the northern region by 4 °C and by 5 °C from the southern region.

Following the overview, changes over time in ice conditions were analysed statistically for each individual lake. For this assessment of trends over time, we used the non-parametric Mann–Kendall trend test (Hipel & McLeod 1994), based on yearly mean values for clear and white ice thicknesses across 49 years. As the detailed ice monitoring programme terminated more than a decade ago, we extrapolated trends until 2023 using the Theil-Sen method (Sen 1968). This method relies on the median of all slopes and intercepts between two subsequent points to determine a median slope in a statistically significant trend. To obtain total ice thickness, the trends for clear and white ice were extrapolated separately and summed keeping the statistically determined trends. If a trend was not statistically significant at *p* > 0.05, the mean value across 1960–2009 was used to gain an estimate for the non-significant ice structure. The extrapolations were then compared to ice safety guidelines where total lake ice thickness below 15 cm was considered unsafe when discussing public lake ice safety.

**Fig. 3:**
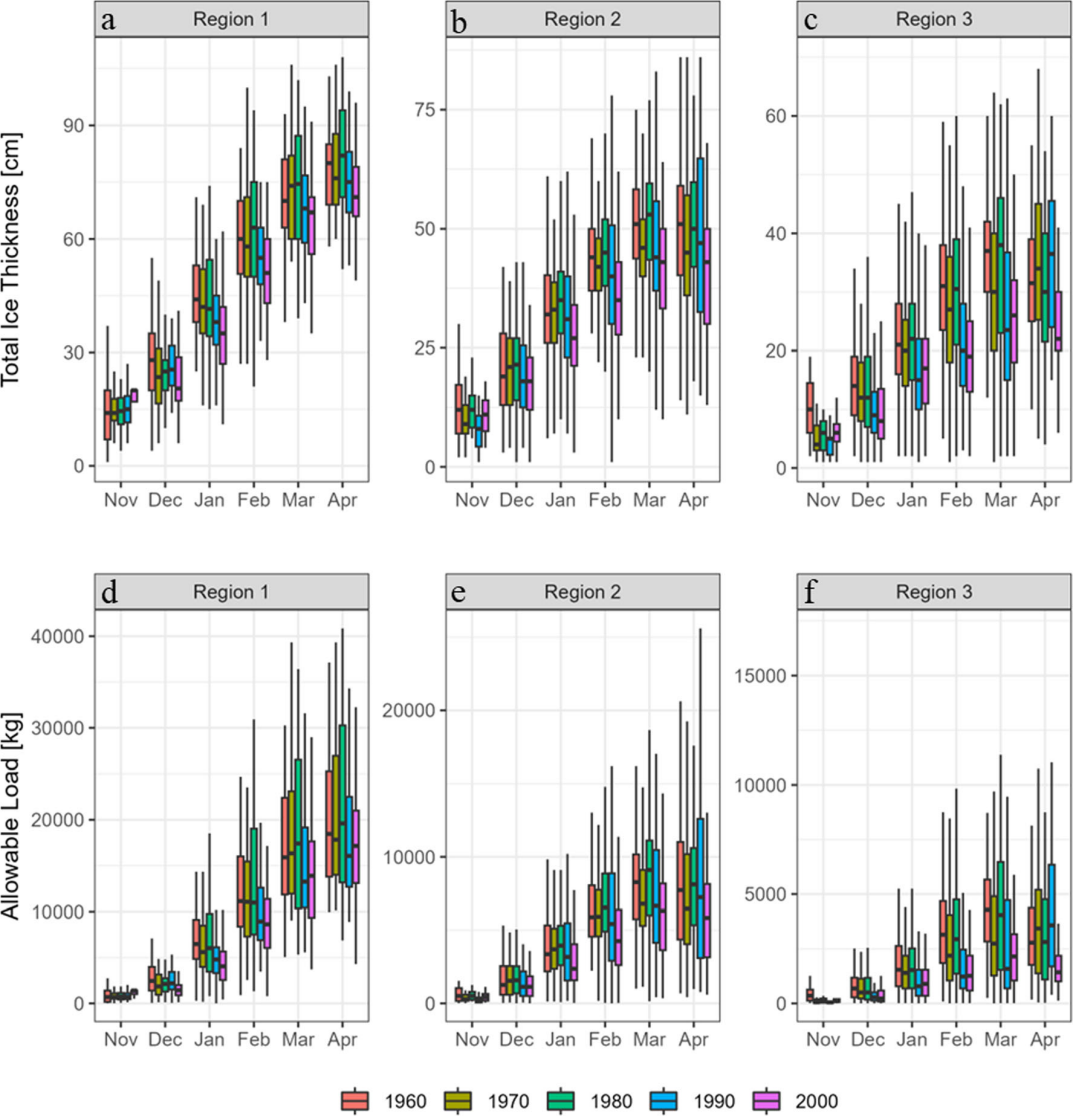
Box plots, grouped by month, of observed total ice thickness, **a** Region 1 (< − 10 °C) ice thickness in cm, **b** Region 2 (− 5 to − 10 °C) total ice thickness in cm, **c** Region 3 (> − 5 °C) total ice thickness in cm and estimated allowable loads, using Eq. 2, **d** Region 1 (< − 10 °C) allowable loads in kg, **e** Region 2 allowable loads (− 5 to − 10 °C) in kg, **f** Region 3 (> − 5 °C) allowable loads in kg for 21 study lakes. Allowable loads estimated using Eq. 2. The data are divided into three regions (see Fig. 2) and over five decades (49 years) starting from November the year before the start of each decade

Finally, to test the second hypothesis simple linear regressions on log-transformed data was applied. All data inspection, data handling and data analyses were conducted in R-statistics (version 4.3.1, R Core Team 2023) and QGIS (version 3.22.7, QGIS Development Team 2009). When a statistically significant difference is mentioned, we report p-values below 0.05. For the overview data (i.e. Figure 3) statistics was not performed due to high total errors when grouping data over regions and decades.

## Results

### General patterns in ice cover dynamics over five decades

**Fig. 4:**
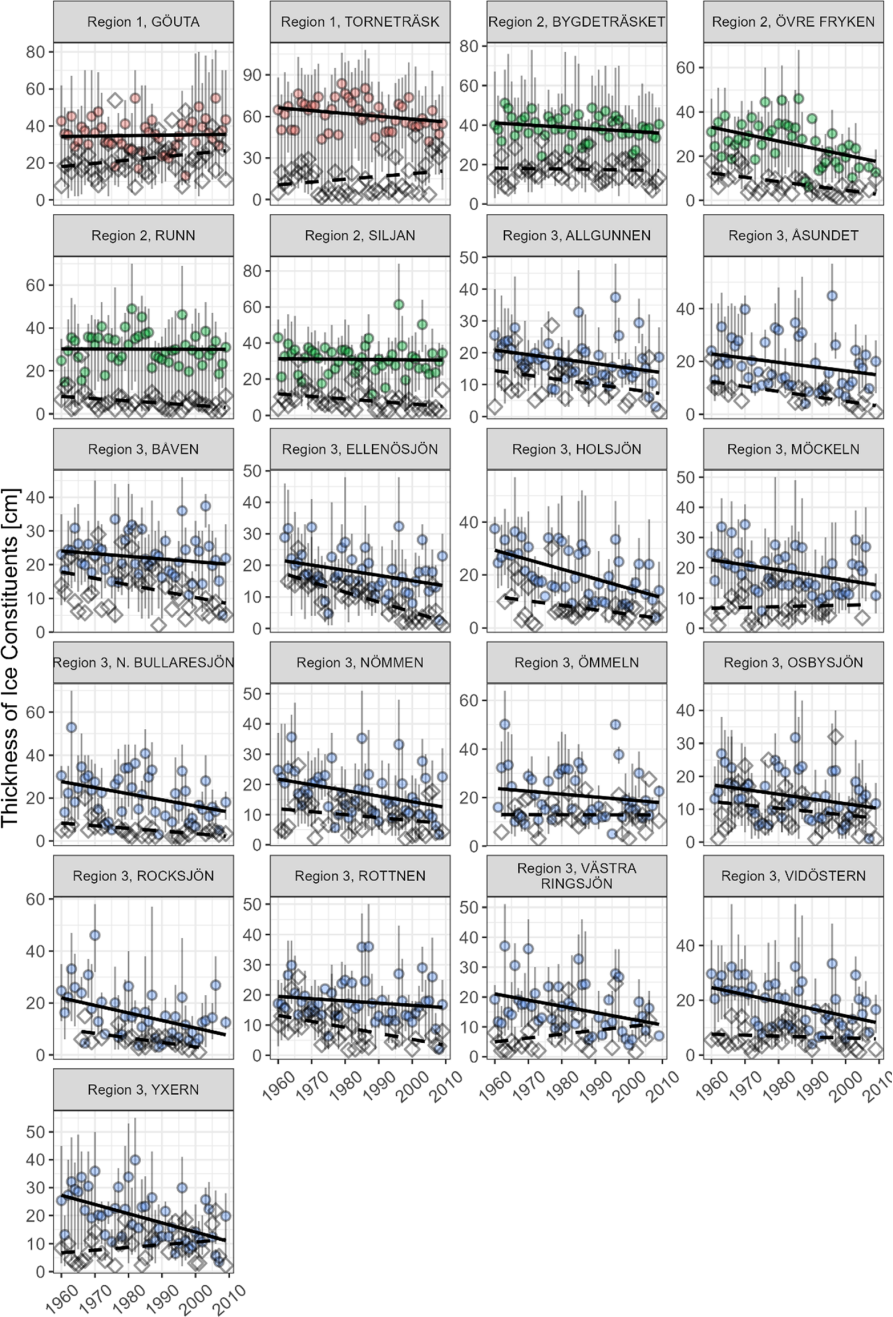
The thickness of clear ice (circles and solid line for clarification) and the thickness of white ice (diamonds and dashed line for clarification) in each of the 21 lakes. Colours indicate the regions, i.e. red for the northern region, green for the central region and blue for the southern region. Bars show upper limit and lower limit of yearly ice thickness. Note, lines show direction of trends regardless of statistical significance. Slopes and statistical significances are presented in Table 2

The overview of the data revealed a decline in the total ice thickness in all three regions. The smallest decline observed was 3–8 cm per decade in the northern and central regions, while the highest rates of decline occurred in the southern region where ice thickness was reduced by 4–12 cm per decade (Fig. 3). Lake ice became thinner during all months when moving closer to present day with February having a peak loss in ice thickness, which again was most pronounced in southern region (Fig. 3). The southern region also indicated earlier deterioration than the other two regions as the April value was already below that of the previous month while the other two regions reached a plateau in April. Total thickness of lake ice was highest during the 1960s and decreased towards present day (Fig. 3) and varied between 4.5 and 126.5 cm between all the lakes, with a mean total ice thickness of 37 cm. In 16 out of the 21 lakes, total ice thickness significantly decreased (Table 2) and the estimated to an average loss 0.22 ± 0.03 cm yr^−1^ (standard error, *p* < 0.05) across Sweden during 1960 to 2009. Yet, the vast majority of lakes were still regarded safe for wintertime activities as they commonly reached a total ice thickness within of 15 cm (Fig. 4) corresponding to ice safety guidelines (Minnesota Department of Natural Resources 2022; Issäkerhetrådet 2012; Swedish Snowmobile Owners State Organisation 2012; Canadian Red Cross 2014).

**Table 2:** Results from Mann–Kendall trend test and Sen slopes for the thickness of total ice, clear- and white ice over time in the 21 study lakes, ranging from 1960 to 2009. The individual range of years for each lake is reported in Table 1. A p-value < 0.05 was considered statistically significant. Significant relationships are indicated in black, non-significant relationships faded to grey. Data ordered from warmest to coldest region

Lake	Trend over time in clear ice thickness			Trend over time in white ice thickness			Trend over time in total ice thickness		
	*p*-value	Sen slope	Tau	*p*-value	Sen slope	Tau	*p*-value	Sen slope	Tau
Rocksjön	0.01	− 0.27	− 0.28	0.02	− 0.14	0.36	< 0.01	− 0.31	− 0.35
Ellenösjön	0.02	− 0.17	− 0.25	< 0.01	− 0.32	− 0.62	< 0.01	− 0.39	− 0.33
Osbysjön	0.06	− 0.14	0.09	0.14	− 0.11	− 0.16	0.06	− 0.23	− 0.19
Holsjön	< 0.01	− 0.37	− 0.41	0.11	− 0.1	− 0.19	< 0.01	− 0.42	− 0.35
Ömmeln	0.56	− 0.05	< − 0.01	0.87	− 0.02	− 0.025	0.89	< 0.01	0.02
N. Bullaresjön	0.02	− 0.27	− 0.24	< 0.01	− 0.08	− 0.36	0.01	− 0.36	− 0.26
Allgunnen	< 0.01	− 0.18	− 0.30	0.06	− 0.15	− 0.247	< 0.01	− 0.30	− 0.33
V. Ringsjön	0.02	− 0.22	− 0.26	0.19	0.09	0.197	0.01	− 0.26	− 0.26
Yxern	< 0.01	− 0.35	− 0.39	0.16	0.10	0.163	0.01	− 0.26	− 0.25
Nömmen	< 0.01	− 0.23	− 0.29	0.09	− 0.11	− 0.189	< 0.01	− 0.21	− 0.29
Rottnen	0.19	− 0.08	− 0.13	0.00	− 0.19	0.24	< 0.01	− 0.35	− 0.31
Åsundet	0.1	− 0.16	0.04	< 0.01	− 0.20	− 0.37	0.03	− 0.23	− 0.23
Vidöstern	< 0.01	− 0.27	− 0.31	0.39	− 0.04	− 0.10	< 0.01	− 0.31	− 0.29
Möckeln	0.01	− 0.19	− 0.27	0.61	0.04	0.07	< 0.01	− 0.30	− 0.28
Båven	0.15	− 0.09	− 0.14	0.01	− 0.22	− 0.27	< 0.01	− 0.30	− 0.28
Bygdeträsket	0.12	− 0.1	− 0.16	0.54	− 0.04	− 0.00	0.11	− 0.12	− 0.16
Övre Fryken	< 0.01	− 0.31	− 0.30	< 0.01	− 0.18	− 0.41	< 0.01	− 0.48	− 0.42
Runn	0.96	− 0.01	− 0.00	0.02	− 0.06	− 0.24	0.14	− 0.12	− 0.15
Siljan	0.40	− 0.08	− 0.00	0.01	− 0.13	− 0.28	0.02	− 0.23	− 0.22
Göutan	0.96	0.01	0.05	0.05	0.18	0.19	0.03	0.19	0.01
Torneträsk	0.04	− 0.23	− 0.2	0.27	0.09	0.109	0.95	− 0.02	− 0.01

To further investigate this decline, the ice structure was studied separately within each lake. Clear ice layer thickness varied between 1 and 108 cm, with a mean of 25 cm across the 21 Swedish lakes while white ice ranged from 1 to 67 cm, with a mean of 10 cm (Fig. 4). In 12 of the 21 lakes the thickness of the clear ice layer significantly declined (*p* < 0.05) during the study period, while the white ice layer thickness showed significant declines in nine of the 21 lakes (*p* < 0.05, Table 2, Fig. 4). The extrapolation analysis showed an increasing number of unsafe lakes, i.e. not reaching a total ice thickness of 15 cm. The total lake ice thickness was below the ice safety guideline in four lakes in 2009 which increased to nine lakes becoming unsafe in 2023.

### Regional patterns of lake ice structure

The northern region had observations from two lakes, i.e. Torneträsk and Göutan. These two lakes had dissimilar patterns. Torneträsk showed a statistically significant decline (*p* < 0.05) in the thickness of the clear ice layer with an average decline of − 0.23 cm yr^−1^ (1960–2009, Table 2), while Göutan exhibited a statistically significant increase (*p* < 0.05) in the thickness of the white ice layer of 0.18 cm yr ^−1^ (1960–2009, Table 2). Both lakes remained above 50 cm in total ice thickness, indicating high but varying allowable loads.

In the four lakes in the central region of Sweden, a statistically significant decline (*p* < 0.05) in the thickness of clear ice was found in lake Övre Fryken with an average decline of − 0.31 cm yr^−1^ across the study period (Table 2). This lake also showed a decline of − 0.17 cm yr^−1^ (*p* < 0.05) in the thickness of the white ice layer. This loss of white ice was mimicked in lake Runn and Siljan with white ice declines of − 0.06 cm yr^−1^ and − 0.13 cm yr^−1^ (*p* < 0.05) but neither of these had declines in clear ice (Table 2). Övre Fryken had reached a clear ice thickness close to 15 cm and a total ice thickness estimated to ~ 20 cm at the end of 2009. Extrapolation of this decline also suggests that this lake has already moved below 15 cm total ice thickness in recent years.

The southern region had statistically significant declines (*p* < 0.05) in 10 out of 15 lakes for clear ice with an average decline of − 0.25 ± 0.03 cm yr^−1^ (standard error, *p* < 0.05) and a maximum decline in clear ice of − 0.37 cm yr^−1^ (*p* < 0.05) for Holsjön. Out of the 15 lakes studied, three showed concurrent significant declines (*p* < 0.05) in both clear and white ice. However, a total of six lakes showed statistically significant declines (*p* < 0.05, Table 2). The lake with the strongest overall decline in ice thickness, Ellenösjön, was in the southern region with − 0.32 cm yr^−1^ (*p* < 0.05, Table 2). In this region, extrapolation to 2023 revealed an increasing number of lakes where four lakes were below the 15 cm total ice thickness guideline in 2009 moving to eight lakes below this threshold in 2023.

### Lake ice safety estimates

Lake ice safety was assessed by determining the allowable load on ice (Eq. 2), which varied from 1.75 kg to more than 40 tons, with an average of 6319 kg across the 21 lakes during 1960–2009 (Fig. 3). Peak allowable loads usually occurred in March for the southern and central region while a peak with more than 40 tons allowable load due to 108 cm of pure clear ice conditions was observed in April in the northern region. Comparing allowable loads from the first decade (1960–1969) and the last decade (2000–2009) showed a decline in the allowable load in the southern region by 55% on average, where the early months (November–December) were most affected. The decline in the allowable load was much less pronounced in the other two regions (Fig. 3). Comparing Eqs. 1 and 2 lead to a substantially lower allowable load of up to 50% when white ice conditions were taken into consideration, i.e. when Eq. 2 was applied (Fig. S2).

### Lake ice structure and temperature

**Fig. 5:**
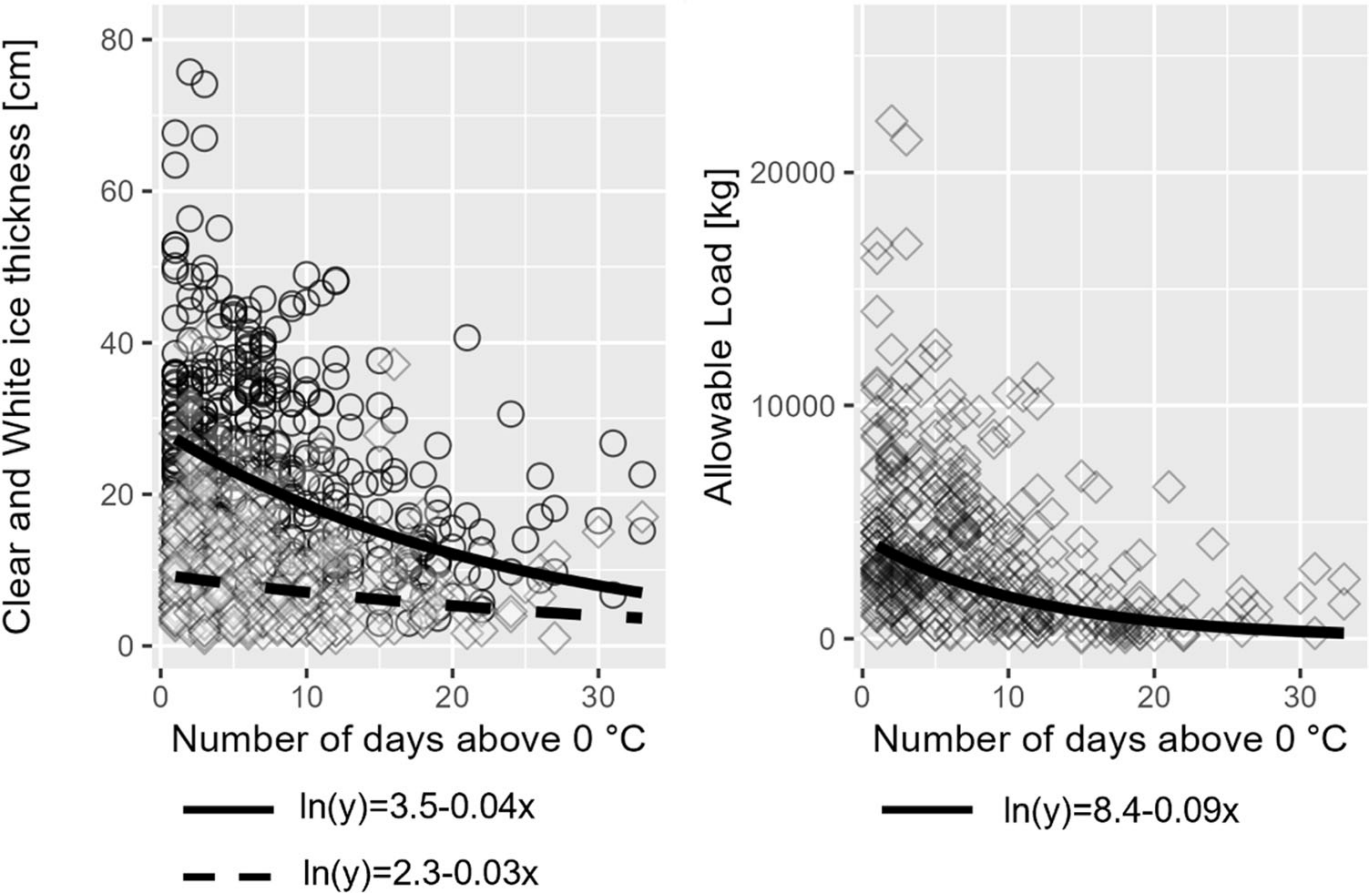
Natural logarithmic relationships between the number of days when daily mean air temperatures remain above 0 °C during the ice cover period and **a** the thickness of clear ice (circles and solid line) and white ice (diamonds and dashed line; left panel) and **b** the allowable load estimated using Eq. 2. All regressions are significant with *p* < 0.05

We found that the number of days when daily mean air temperatures stayed above the freezing point during the ice cover period was significantly related to both the thickness of the clear ice layer and the thickness of the white ice layer (*p* < 0.05, Fig. 5a). An increasing number of days with T > 0 °C during the ice cover period caused a particularly rapid decline in the thickness of the clear ice layer which in turn resulted in a rapid decrease in the estimated allowable load on ice (*p* < 0.05, Fig. 5b).

## Discussion

### Regional differences in lake ice sheets

We observed most pronounced changes in total ice thickness as well as the thickness of clear ice in the southern part of Sweden. This was the case both when lakes were studied individually across the years (1960–2009), and across the five decades studied regionally (Fig. 3). Lake depth and morphometry showed no clear patterns across lakes and regions in lake ice structure. Most declines observed in this study fall within, but at the lower range, of lake ice thickness declines reported in literature, ranging from − 0.10 to − 2.5 cm yr^−1^ (Korhonen 2006; Imrit et al. 2022; Li et al. 2022; Stefanidis et al. 2022). In contrast to the Swedish lakes studied here, Korhonen (2006) found many Finnish lakes to have increased in ice thickness over time, whereas we had only one statistically significant increase in in total ice thickness, mainly due to white ice increases (*p* < 0.05) as clear ice remained stable for lake Göutan located in the north (Table 2). This suggests that Göutan may experience more frequent freeze thaw events promoting white ice build-up than Torneträsk. However, this difference was not discernible when comparing the two lakes for days above T > 0 °C nor in statistical analysis on temperature differences between the two lakes (*p* < 0.05).

The three regions showed differences in which ice structure had most prominent declines, indicating varying responses to changes in climate on ice structure. The two lakes located in the northern region showed opposing patterns in clear and white ice changes over time (Fig. 4, Table 2). Yet, the total ice thickness remained high resulting in stable ice conditions. Within the central region only Övre Fryken had a statistically significant decline in clear ice (Table 2). White ice declines were similar between Övre Fryken and Siljan and Runn, albeit the latter lake showed a 50% smaller trend in white ice thickness (Table 2). This suggests that the central region is moving towards thinner ice, but mainly from freeze thaw events of slush. Additionally, Övre Fryken showed a significant concurrent loss in both clear and white ice and reached below 15 cm when extrapolated to 2023, indicating that select lakes in the central region are moving towards that of the trends in the southern region. Like Övre Fryken significant decreasing trends in clear ice (*p* < 0.05) with three concurrent declines in both clear and white ice was found in the south (Table 2). This suggests that the southern region is experiencing clear ice loss, while the three lakes where only white ice declines were statistically significant are still in similar conditions as Övre Fryken.

### Warmer air temperature as a driver for ice structure changes

Air temperature as a main driver for ice cover changes is well known (Korhonen 2006; Benson et al. 2012; Imrit & Sharma 2021). We related warmer air temperatures to ice structure changes by counting the number of days when daily mean air temperatures stay above the freezing point during the ice cover period (T > 0 °C). This approach includes a time component and a measure of freezing and thawing cycles during the ice cover period, which has been shown to be important for the formation of clear and white ice (Weyhenmeyer et al. 2022). We suggest that the number of days T > 0 °C is a suitable predictor for ice structure (Fig. 5a). Particularly sensitive to the number of days with T > 0 °C was the thickness of the clear ice layer which rapidly declined with an increasing number of days with T > 0 °C (*p* < 0.05, Fig. 5a). This resulted in marked changes in allowable loads past five days T > 0 °C (Fig. 5b).

We recommend that future studies focus on detailed analyses of T > 0 °C during the ice cover period and to further investigate how fluctuations in T > 0 °C affect the pattern of ice structure, i.e. the ice thickness of clear and white ice in lakes. This should include detailed studies on precipitation and snow, a pre-requisite for white ice formation not studied here, on lake ice alongside fluctuations in T > 0 °C. The timeline of when T > 0 °C occurs may also help explain the complexity of ice-on and ice-off dynamics observed by Newton & Mullan (2021). The lakes showing a shift in ice-on dates occurring earlier may be located in areas where T > 0 °C occurs later in the season while those lake that show a decrease in both ice on and off-dates may have a more equal distribution of days T > 0 °C. Consecutive days with T > 0 °C should also have a more pronounced effect on the ice structure compared to several single days with T > 0 °C during the ice cover period. The effect can further be reinforced depending on the amount of snow on ice, precipitation patterns and wind conditions (Leppäranta 2015).

### Lake ice safety—a function of ice structure

Lake ice safety followed the decline in clear ice reported and was most prominent in the South. In most lakes when clear ice declined white ice either remained stable or decreased at a slower rate. Considering the acceleration in warming trends (World Meteorological Organization 2023), and the sensitivity of clear ice to the number of days with T > 0 °C the results presented here are conservative estimates for current ice conditions. This carries over to allowable loads on lake ice and may be best case scenarios for Swedish lakes. Especially as precipitation is projected to increase, promoting white ice. Not only is white ice weaker than clear ice but increased precipitation in the form of snow reduces the capacity for clear ice to grow further reducing lake ice allowable loads.

**Fig. 6:**
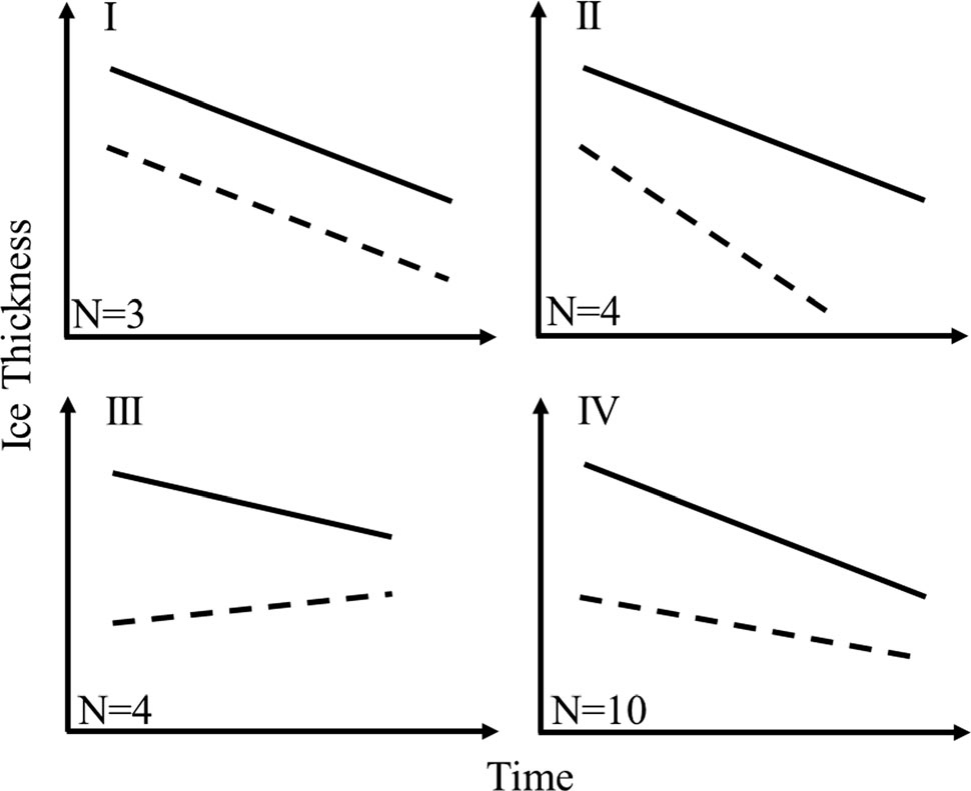
Conceptual figure showing four scenarios of changes over time in the thickness of the clear ice (solid lines) and white ice layer (dashed line). The figure represents the patterns that have been found across 21 Swedish lakes during 1960 to 2009, i.e. ten lakes showed pattern IV, five lakes pattern III, five lakes pattern II and one lake pattern I

Understanding the variations in lake ice structure can help to explain variability in lake ice safety. This is exemplified by the four patterns in clear and white ice observed (Fig. 4) and are illustrated conceptually in Fig. 6. The patterns are: (I) the thickness of clear and white ice show a similar rate of change, (II) the thickness of the white ice layer changes faster than the thickness of the clear ice layer, (III) the thickness of the clear ice layer declines while the thickness of the white ice layer increases over time and (IV) the thickness of the clear ice layer changes faster than the thickness of the white ice layer (Fig. 6). Most common across the study lakes was pattern IV where clear ice declines faster than white ice. This pattern was observed for 10 of the 21 lakes and implies a substantial decline in the future lake ice safety for lakes showing this pattern. The other patterns were less prominent, but pattern III should be considered the most dangerous as this pattern eventually substitutes clear ice with white ice. Yxern and Västra Ringsjön exemplify pattern III (Fig. 4). The remaining patterns I and II are considered less dangerous as these patterns follow total lake ice thickness closer and are covered by current guidelines.

### Future trajectories and public safety

Considering the rapid changes in ice structure presented and discussed here, there is an urgent need in Sweden to restart the monitoring programme of ice structure again to follow changes in lake ice safety and lake ice dynamics. A proper monitoring programme is of particular importance, since the interpretation of satellite images is not yet sufficient to determine the thickness of clear and white ice layers while these can discern ice thickness at a sufficient accuracy (Duguay et al. 2003; Pour et al. 2017; Murfitt and Duguay 2021).

At present it can only be speculated how ice structure has further changed in recent years. For policymakers, this means an added uncertainty in how to deal with safety on lake ice, which has a culturally important place in many countries. With the knowledge that clear ice is decreasing rapidly and that white ice is decreasing slower, later winter months should be considered treacherous as also noted by Sharma et al. (2020). Especially, considering that snowmobiling is a popular and dangerous pastime (Gustafsson & Eriksson 2013) and that other recreational activities in Sweden are common reasons to be exposed to dangerous ice conditions. The majority of unsafe lakes were located in the southern region which is mainly experiencing clear ice loss. Out of nine unsafe lakes, only Övre Fryken was not located in the south and is a lake which is also experiencing clear ice loss. As clear ice is also the more stable ice structure of the two studied and many lakes moving towards further losses in clear ice, the result of this study encourages revision of ice safety guidelines to include clear ice rather than total ice thickness.

## Conclusion

We showed that the structure of lake ice, seldom studied, is rapidly changing in a warmer world. Clear ice carries most of the load on ice and was particularly sensitive to the number of days above where temperatures exceed 0 °C affecting lake ice safety for the public who actively utilises lake ice both recreationally and professionally. To avoid an increase in fatal winter drownings when people fall through unstable ice we recommend to regularly monitor ice structure, to develop better warning systems, to revise ice safety guidelines and to prepare society for large changes in ice safety.

## Supplementary Information

Below is the link to the electronic supplementary material.

Supplementary file1 (PDF 575 KB)
