# Supplementary material for: Rapid lake ice structure changes across Swedish lakes puts public ice safety at risk
Source: Ambio. 2024 Aug 20;54(1):122–34. doi: 10.1007/s13280-024-02067-8 (PMC11607191; doi:10.1007/s13280-024-02067-8)
Supplement: Supplementary file 1 — Supplementary file1 (PDF 575 KB) [file 13280_2024_2067_MOESM1_ESM.pdf]

Ambio

Supplementary Information

Title: **Rapid lake ice quality changes across Swedish lakes puts public ice safety at risk**

Supplementary Figures:

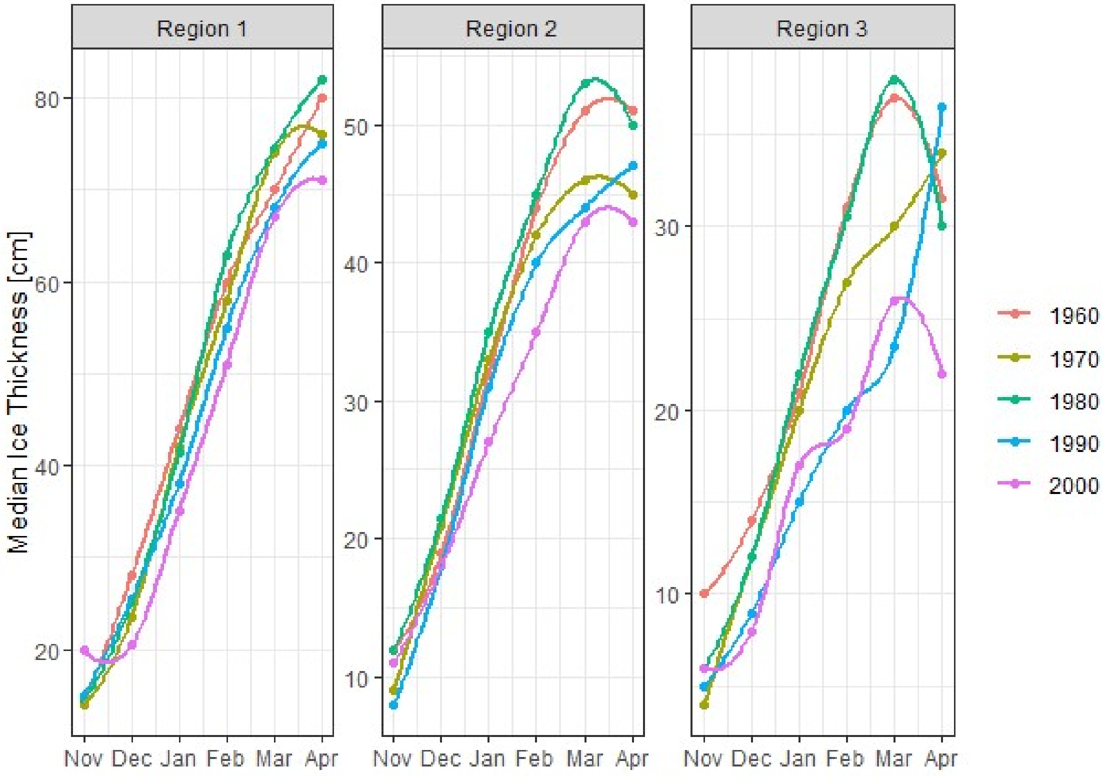

Fig. S1 Median total ice thickness for each month and each decade (indicated with different colors) for three regions in Sweden where Region 1 (< -10 ℃) , Region 2 (-5 ℃ to -10 ℃) Region 3 (>-5 ℃) For the locations of the regions we refer to Fig. 2.

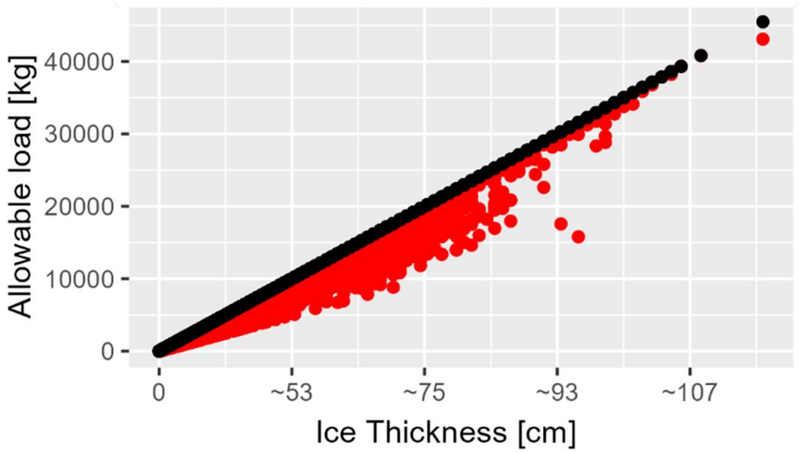

Figure S2. Estimates of the allowable load on ice for each reported ice thickness by using equation 1 (black) and equation 2 (red dots). Note, that equation 1 uses total ice thickness alone and does not distinguish between ice structure types. Equation two uses the percentage of white ice and total ice thickness to estimate allowable loads on ice in kg.

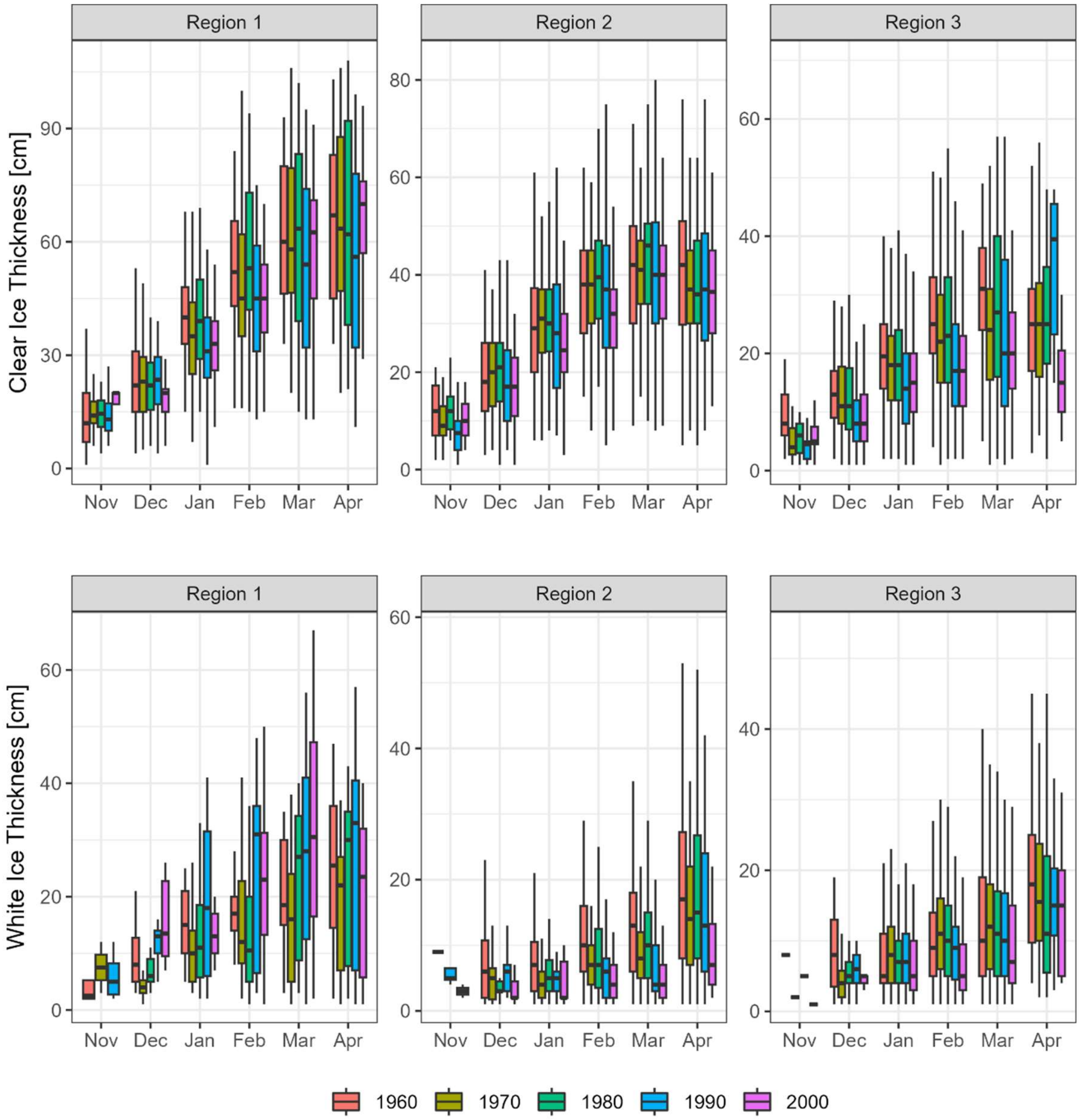

Figure S3. Figure 3. Box plots, grouped by month, of observed clear and white ice thickness for all lakes grouped within assigned regions (see methods) a) Region 1 (< -10 °C) clear ice thickness in cm b) Region 2 (-5 to -10 °C) clear ice thickness in cm c) Region 3 (> -5 °C) clear ice thickness in cm and d) Region 1 (< -10 °C) white ice thickness in cm e) Region 2 (-5 to -10 °C) white ice thickness in cm f) Region 3 (> -5 °C) white ice thickness in cm. Allowable loads estimated using equation 2. The data are divided into three regions (see Fig. 2) and over five decades (49 years) starting from November the year before the start of each decade.
